# Virus-like particle size and molecular weight/mass determination applying gas-phase electrophoresis (native nES GEMMA)

**DOI:** 10.1007/s00216-019-01998-6

**Published:** 2019-07-06

**Authors:** Victor U. Weiss, Ronja Pogan, Samuele Zoratto, Kevin M. Bond, Pascale Boulanger, Martin F. Jarrold, Nicholas Lyktey, Dominik Pahl, Nicole Puffler, Mario Schelhaas, Ekaterina Selivanovitch, Charlotte Uetrecht, Günter Allmaier

**Affiliations:** 1 Institute of Chemical Technologies and Analytics, TU Wien, Getreidemarkt 9/164, 1060 Vienna, Austria; 2 Heinrich Pette Institute, Leibniz Institute for Experimental Virology, Martinistraße 52, 20251 Hamburg, Germany; 3 European XFEL GmbH, Holzkoppel 4, 22869 Schenefeld, Germany; 4 Department of Chemistry, Indiana University, 800 E Kirkwood Ave, Bloomington, IN 47405 USA; 5 Institute for Integrative Biology of the Cell, CEA, CNRS, Université Paris-Sud, Université Paris-Saclay, 91198 Gif-sur-Yvette, France; 6 Institute of Cellular Virology, WWU Münster, Von-Esmarch-Str. 56, 48149 Münster, Germany

**Keywords:** Native nES GEMMA, DMA, VLP, Molecular weight/mass, Size, Mass spectrometry

## Abstract

(Bio-)nanoparticle analysis employing a nano-electrospray gas-phase electrophoretic mobility molecular analyzer (native nES GEMMA) also known as nES differential mobility analyzer (nES DMA) is based on surface-dry analyte separation at ambient pressure. Based on electrophoretic principles, single-charged nanoparticles are separated according to their electrophoretic mobility diameter (EMD) corresponding to the particle size for spherical analytes. Subsequently, it is possible to correlate the (bio-)nanoparticle EMDs to their molecular weight (M_W_) yielding a corresponding fitted curve for an investigated analyte class. Based on such a correlation, (bio-)nanoparticle M_W_ determination via its EMD within one analyte class is possible. Turning our attention to icosahedral, non-enveloped virus-like particles (VLPs), proteinaceous shells, we set up an EMD/M_W_ correlation. We employed native electrospray ionization mass spectrometry (native ESI MS) to obtain M_W_ values of investigated analytes, where possible, after extensive purification. We experienced difficulties in native ESI MS with time-of-flight (ToF) detection to determine M_W_ due to sample inherent characteristics, which was not the case for charge detection (CDMS). nES GEMMA exceeds CDMS in speed of analysis and is likewise less dependent on sample purity and homogeneity. Hence, gas-phase electrophoresis yields calculated M_W_ values in good approximation even when charge resolution was not obtained in native ESI ToF MS. Therefore, both methods-native nES GEMMA-based M_W_ determination via an analyte class inherent EMD/M_W_ correlation and native ESI MS-in the end relate (bio-)nanoparticle M_W_ values. However, they differ significantly in, e.g., ease of instrument operation, sample and analyte handling, or costs of instrumentation.

**Electronic supplementary material:**

The online version of this article (10.1007/s00216-019-01998-6) contains supplementary material, which is available to authorized users.

**Figure:**
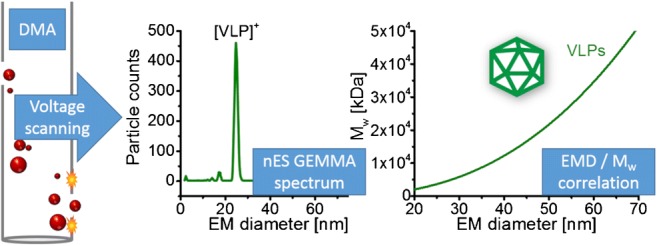
Graphical abstract

## Introduction

Viruses are nanoparticles of biological origin: A proteinaceous capsid protects the viral genome from the exterior. Additional protection can be conveyed by a lipid membrane, which is additionally modified by (glyco-) proteins to enable attachment to target cells (e.g., [1]). Only upon target cell infection, the genomic material of the virus is intended for release. This concept is of interest for pharmacological applications as virus bionanoparticles can be interpreted as carriers enabling the shielded, targeted transport of cargo material. Alternatively, viral particles without any encapsulated cargo can be employed for vaccination. In both cases, corresponding particles are referred to as virus-like particles (VLPs) (e.g., [2]).

To allow for VLP application in the field of pharmaceutics, their thorough characterization and preparation batch control, e.g., in terms of particle homogeneity, purity of preparations, particle size, and molecular weight (M_W_), is of importance. For the latter, native electrospray ionization mass spectrometry (native ESI MS) mostly in combination with time-of-flight (ToF) analyzers evolved as method of choice, yielding M_W_ values of bionanoparticles after deconvolution of mass spectra. Such, the M_W_ of dimorphic hepatitis B-based VLPs could be obtained already in 2008 [3]. In addition, several other VLPs or subviral particles [4, 5] up to a maximum M_W_ of 17.9 MDa with charge state assignment [6] or employing cryodetection with matrix assisted laser desorption/ionization time-of-flight (MALDI TOF) MS [7] could be investigated. ESI with charge detection mass spectrometry (CDMS) [8, 9] even allowed detection of VLPs up to 26.8 MDa, employing bacteriophage P22 as model [10, 11]. Besides enabling the analysis of VLPs, also capsid binding to antibody fragments [12], pH-dependent VLP decomposition [13] or the investigation of VLP capsid assembly [14] was accessible via native ESI MS. However, none of these measurements can be carried out on standard commercial instruments. Employed mass spectrometers are usually customized in terms of, e.g., applied pressures, employed carrier gas or voltage settings, or pose completely new instrumental developments. In 2018, for instance, Dominguez-Medina and coworkers reported the analysis of bacteriophage T5 icosahedral capsids either in their empty VLP form (M_W_ ~ 27 MDa) or in their DNA-filled native form (M_W_ ~ 108 MDa) [15]. For these experiments, nanochemical resonators were employed [16, 17].

As alternative, M_W_ determination can also be based on gas-phase electrophoresis data employing a nano-electrospray gas-phase electrophoretic mobility molecular analyzer (native nES GEMMA also known as nES differential mobility analyzer, nES DMA) [18]. Bionanoparticles are electrosprayed from a volatile, aqueous electrolyte solution. Subsequently, droplets are dried. At the same time, charge equilibration occurs in a bipolar atmosphere induced by, e.g., an α-particle emitter like ^210^Po, an alternating corona discharger or a soft X-ray tube [19, 20]. Hence, singly charged particles are obtained (besides a majority of neutral objects), which are then separated according to electrophoretic principles in the gas phase at ambient pressure. A particle charge of one leads to bionanoparticle separation according only to the surface-dry particle size (electrophoretic mobility diameter, EMD) in a high laminar sheath flow of particle-free, dried air, and a tunable electric field. Variation of the field strength enables size separation of sample components. The obtained monodisperse aerosol is subsequently introduced to the detector unit of the instrument (ultrafine condensation particle counter, CPC), where bionanoparticles act as condensation nuclei in a supersaturated atmosphere of either n-butanol or water. Obtained droplets are counted as they pass a focused laser beam. Such an instrumentation has previously been employed for the analysis of liposomes [21, 22], exosomes [23], viruses [24–29], proteins and protein aggregates [30], polysaccharides [31–33], DNA [34], polymers [35–37], and nanoparticles in general [38–42]. Besides yielding information on analyte surface-dry particle size and size distribution, particle number concentration detection is possible in accordance with recommendations of the European Commission for characterization of nanoparticle material (2011/696/EU from October 18^th^, 2011). Furthermore, analytes can be size-selected for further analyses employing orthogonal methods for instance electron microscopy [43], atomic force microscopy [44], spectroscopic techniques [45, 46], or antibody-based nanoparticle recognition [44, 47]. It is of note that LiquiScan ES, MacroIMS, and SMPS are synonyms of the same instrument found in literature.

Furthermore, as first demonstrated in great detail by Bacher and colleagues [48] for proteins, obtained EMD results can be related to particle M_W_ values yielding a corresponding correlation. Hence, based on a protein EMD value, its M_W_ can be calculated in good approximation. Similar approaches have been demonstrated, e.g., for intact viruses [4] and polysaccharides [32]. It is of note that for the latter two, the obtained EMD/M_W_ correlations deviated from the protein case. For instance for polysaccharides, it was reasoned that additional factors might influence the EMD/M_W_ correlation, inter alia particle shape, or insufficient characterization of applied, commercially available standards. Likewise, for elongated virus structures (tobacco mosaic virus), effects like bending of analytes due to surface effects of droplets generated during the nES process were observed [49].

In the current manuscript, we focus on nano-objects, namely VLPs, which are approximately spherical (icosahedral) and non-enveloped (empty protein shells). We asked ourselves, if a native nES GEMMA-based EMD/M_W_ correlation for VLPs is likewise differing from correlations described for other classes of analytes. In addition, we wanted to investigate if a corresponding EMD/M_W_ correlation will allow an approx. M_W_ determination of this class of bionanoparticles with a relatively quick, cheap (~ 80.000 €), less challenging (in terms of sample quality) and easy to handle analytical setup in comparison to native ESI MS. Mind however, that a high accuracy M_W_ determination of VLPs is only possible on the basis of MS-derived data. Hence, both methods have to be regarded as yielding complementary information in terms of analyte size and M_W_, sample quality, and particle number concentration.

## Materials and methods

### Chemicals

Ammonium acetate (NH_4_OAc, ≥ 99.99%) and ammonium hydroxide (ACS reagent) were both purchased from Sigma-Aldrich (Steinheim, Germany).

### Electrolyte

NH_4_OAc, 40 mM ammonium acetate at pH 7.0 was used as electrolyte solution for desalting of VLPs and for sample dilution for nES GEMMA and ESI MS. Electrolyte solutions for CDMS measurements are detailed with corresponding experiments. NH_4_OAc solution was filtered (0.2 μm pore size syringe filters, Sartorius, Göttingen, Germany) prior application. Millipore (Billerica, MA, USA) grade water was employed (18.2 MΩcm resistivity at 25 °C).

### Biological material

Norovirus West Chester GI.1 VLPs (3 mg/mL in PBS, pH 7.4) were produced in insect cells and kindly provided by Grant Hansman, Heidelberg, Germany, and CPMV VLPs (4 mg/mL in 10 mM sodium phosphate, pH 7.0) were from John Innes Centre (kindly provided by George Lomonossoff, Norwich, UK). Bacteriophage P22 VLPs (2 mg/mL in 50 mM sodium phosphate, pH 7.0 including 100 mM sodium chloride and 200 ppm sodium azide) were obtained from Indiana University Bloomington (Bloomington, IN, USA), bacteriophage T5 VLPs (0.3 mg/mL, i.e., 7 × 10^12^ empty capsids/mL in PBS) from the Institute for Integrative Biology of the Cell (I2BC), Gif-sur-Yvette, France). Human papillomavirus type 16 (HPV16, 0.3 mg/mL in PBS additionally including 625 mM sodium chloride, 0.9 mM calcium chloride, 0.5 mM magnesium chloride, and 2.1 mM potassium chloride) VLPs were prepared from mammalian cells as previously described [50].

### Instrumentation

Gas-phase electrophoresis was performed on two setups: a nES GEMMA instrument (TSI Inc., Shoreview, MN, USA) consisting of a nES aerosol generator (model 3480) including a ^210^Po α-particle source, an electrostatic classifier (model 3080) with a nano-differential mobility analyzer (nDMA) and an n-butanol-based ultrafine condensation particle counter (model 3025A or 3776C) was applied as instrument A. Instrument B consisted of a model 3482 nES aerosol generator including a soft X-ray source, an electrostatic classifier (model 3082) and a water-based ultrafine condensation particle counter (model 3788). Twenty-five µm inner diameter polyimide coated fused silica capillaries (Polymicro, obtained via Optronis, Kehl, Germany) with in-house made tips [51] were employed to transfer analytes from the liquid to the gas phase. Settings for a stable Taylor cone at the nES tip were chosen, typically around 2 kV voltage resulting in approx. − 375 nA current, 0.1 liters per minute (Lpm) CO_2_ (Messer, Gumpoldskirchen, Austria) and 1.0 Lpm filtered, dried ambient air. Four pounds per square inch differential (psid, approx. 27.6 kPa) were applied to additionally move the sample through the capillary. Fifteen Lpm sheath flow filtered ambient air was used to size-separate VLPs in an EMD range from 2 to 65 nm. The corresponding EMD size range was scanned for 120 s. Subsequently, the applied voltage was adjusted to starting values within a 30-s timeframe. Seven datasets (raw data obtained from instrument software, MacroIMS manager v2.0.1.0) were combined via their median to yield a corresponding spectrum. Lastly, Gaussian peaks were fitted to spectra via Origin software (OriginPro v9.1.0) to obtain EMD values.

HPV16 VLPs cannot be produced in high yields. Due to the resulting low VLP concentration, samples were only analyzed between 30 and 65 nm EMD to increase the scanning time in this range. In addition, a 40 μm inner diameter capillary was employed to reduce the surface to volume ratio and hence the probability of analyte loss due to VLP interaction with the fused silica material of the nES capillary. This resulted in significantly higher particle numbers detected per channel and hence a discernible VLP peak.

For CPMV and P22 VLPs, native MS was performed on a Q-Tof 2 instrument (Waters, Manchester, UK, and MS Vision, Almere, the Netherlands) modified for high mass experiments [52, 53] and calibrated with cesium iodide. A nano-ESI source in positive ion mode with a source pressure of 10 mbar was used. Capillaries were produced in-house. Borosilicate glass tubes (inner diameter 0.68 mm, outer diameter 1.2 mm with filament; World Precision Instruments, Sarasota, FL, USA) were pulled using a two-step program in a micropipette puller (model P-1000 from Sutter Instruments, Novato, CA, USA) with a squared box filament (2.5 × 2.5 mm). Subsequently, the capillaries were gold-coated using a sputter coater (Quorum Technologies, East Sussex, UK, 40 mA, 200 s, tooling factor of 2.3 and end bleed vacuum of 8 × 10^−2^ mbar argon) and opened directly on the sample cone of the mass spectrometer. Voltages of 1.45–1.65 kV and 145–155 V were applied to the capillary and cone, respectively. Xenon (purity 5.0) was used as collision gas at a pressure of 1.7–2.0 × 10^−2^ mbar to improve the transmission of large ions [53]. Collision energies were ramped from 10 to 400 V. MS profile and repetition frequency of the pusher pulse were adjusted to high mass range. Mass spectra were analyzed using MassLynx (Waters, Manchester, UK).

Mass spectra of norovirus West Chester VLPs were obtained using a home-built ESI CDMS system, described in detail elsewhere [54]. Briefly, CDMS is a single particle MS technique, which retrieves *m/z* and *z* for each ion allowing direct mass determination from a charge conducting cylinder functioning also as electrostatic ion trap. Hence, masses of large, heterogeneous biological complexes can be measured. An automated nano-ESI source (Advion, Ithaca, NY, USA) was used to generate ions with a capillary voltage of 1.7 kV. The ions then enter a heated metal capillary and are transmitted using various ion optics to a dual hemispherical deflection energy analyzer which selects ions with energies centered on 100 eV/*z*. These ions then enter a modified cone trap. Here, trapped ions oscillate back and forth in a charge detection cylinder for 100 ms. Mass spectra were generated by binning of the single ion masses. Spectra were subsequently analyzed by fitting Gaussian peaks with Origin software (OriginPro 2016).

### Sample preparation

In order to enable native nES-based analysis of VLPs, employed storage buffers (often including additional salt components or stabilizing agents) had to be replaced by a volatile electrolyte solution. Else, these additional sample components were shown to lead to an increased peak heterogeneity of the analytes of interest and, in nES GEMMA, an elevated baseline resulting from clustering of small, non-volatile molecules during the nES process [55]. As in previous studies, we opted for ammonium acetate and carried out removal of small, buffer-associated sample components via spin filtration [42] employing 10 kDa M_W_ cutoff filters (Vivaspin-polyethersulfone membrane, Vivacon-regenerated cellulose membrane-both from Sartorius or centrifugal filters-polyethersulfone membrane, VWR, Vienna, Austria). Between 3 and 5 filtration steps were necessary to remove non-volatile additional sample components. Sample concentration for measurements was typically well below 1 mg/mL protein content (based on originally determined values and sample dilution).

## Results and discussion

It was the aim of our investigation to analyze VLP material with gas-phase electrophoresis on a native nES GEMMA instrument and to compare results with data obtained from native ESI MS and from literature. We intended to setup an EMD/M_W_ correlation for VLPs to allow for future VLP M_W_ determination based on gas-phase electrophoresis directed towards analysis of samples not applicable to native ESI ToF MS.

### Native nES GEMMA analysis of VLPs

**Fig. 1:**
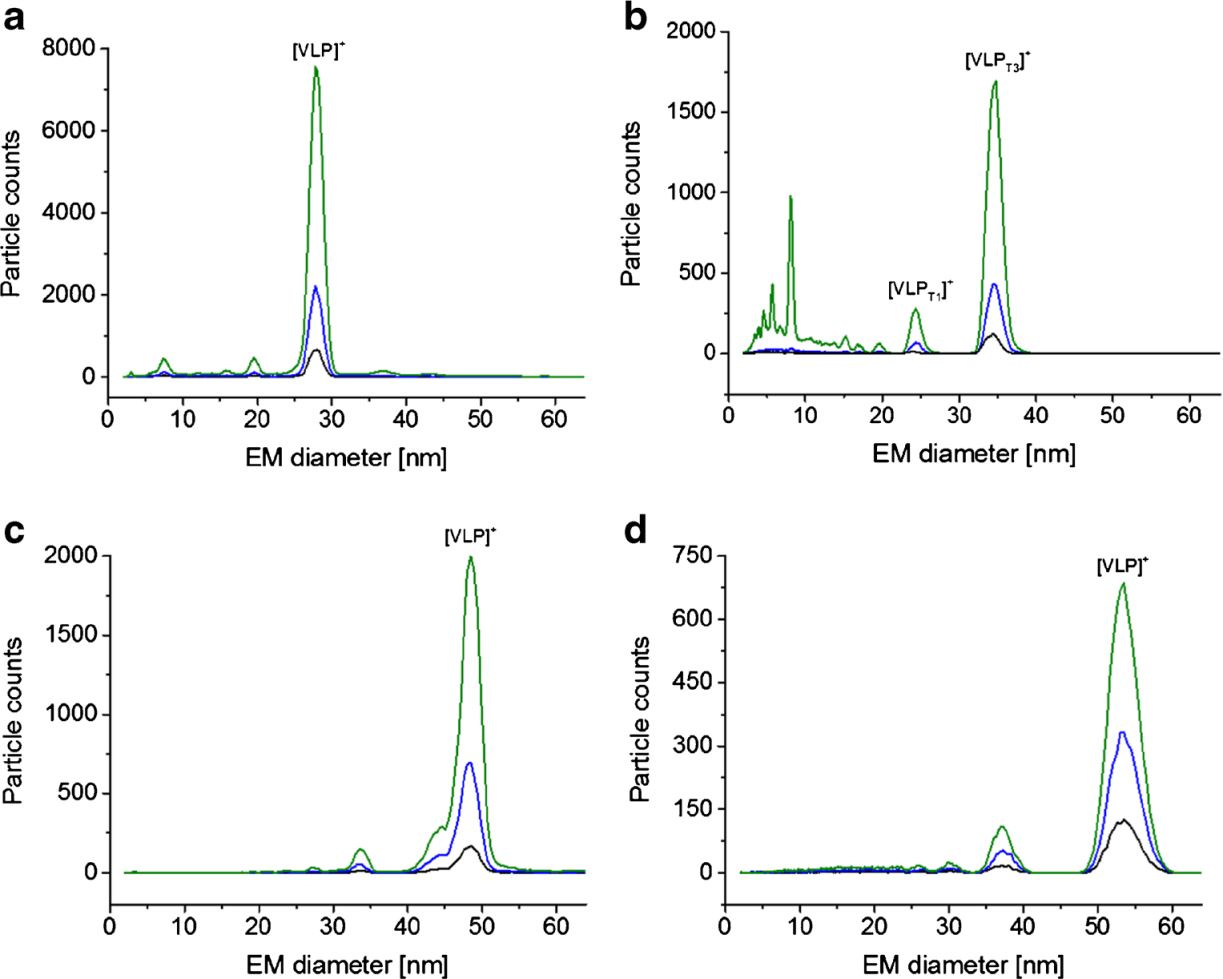
nES GEMMA data for VLPs: CPMV (**a**), norovirus West Chester (**b**), bacteriophage P22 (**c**), and bacteriophage T5 capsids (**d**). All VLPs are shown in three different dilutions of obtained material after solution exchange to 40 mM NH_4_OAc, pH 7.0 employing 10 kDa M_W_ cutoff membrane filters. (Typically, overall dilutions were in the range of 1:10 to 1:250 [*v*:*v*] of the original VLP stock solutions resulting from solution exchange and sample dilution steps)

In previous work, we had already described the analysis of VLP or VLP-like material based on hepatitis B virus capsids (HBV) with two icosahedral symmetries [12] and subviral particles of a human rhinovirus serotype (HRV-A2) [4]. We now turned to further VLP material and analyzed bionanoparticles derived from cowpea mosaic virus (CPMV), a norovirus strain and particles from bacteriophage P22 and T5 via gas-phase electrophoresis. Figure 1 depicts corresponding native nES GEMMA spectra. An overview of investigated VLPs and resulting EMD values are listed in Table 1. In order to exclude the possibility of unspecific aggregation of sample components, VLPs were electrosprayed from samples diluted to at least three different concentrations. Unspecific aggregation of components was excluded, if peaks remained at identical EMD values for all investigated dilutions.

**Table 1:** Overview on investigated VLP material as well as data taken from literature as indicated

	VLP	EM diameter (nm)		Based on	M_W_ (kDa)		Based on
1	Norovirus West Chester T1 VLP	*24.22*	*± 0.21*	*–*	*3320*	*± 30*	*CDMS*
2	Hepatitis B virus (HBV) T3 VLP	24.22	± 0.40	[12]	3004	± 3	MS [12]
3	Hepatitis B virus (HBV) T4 VLP	26.84	± 0.44	[12]	4006	± 3	MS [12]
4	Cowpea mosaic virus (CPMV) VLP	*27.88*	*± 0.04*	**–**	3940	± n.a.	[56]
5	Subviral B particle of human rhinovirus 2	28.68	± 0.07	[4]	5210	± 2	MS [4]
6	Norovirus West Chester T3 VLP	*34.47*	*± 0.15*	*–*	*10,260*	*± 40*	*CDMS*
7	Bacteriophage P22 VLP	*48.44*	*± 0.12*	**–**	19,840	± n.a.	MS [11]
8	Bacteriophage T5 VLP	*53.45*	*± 0.09*	**–**	27,200	± 2300	MS [15]

New data is presented in italics. An exemplary CDMS spectrum of investigated VLPs is shown in the ESM (Fig. S1). At least *N* = 3 technical replicates were used per EMD value. Errors provided are standard deviations

**Fig. 2:**
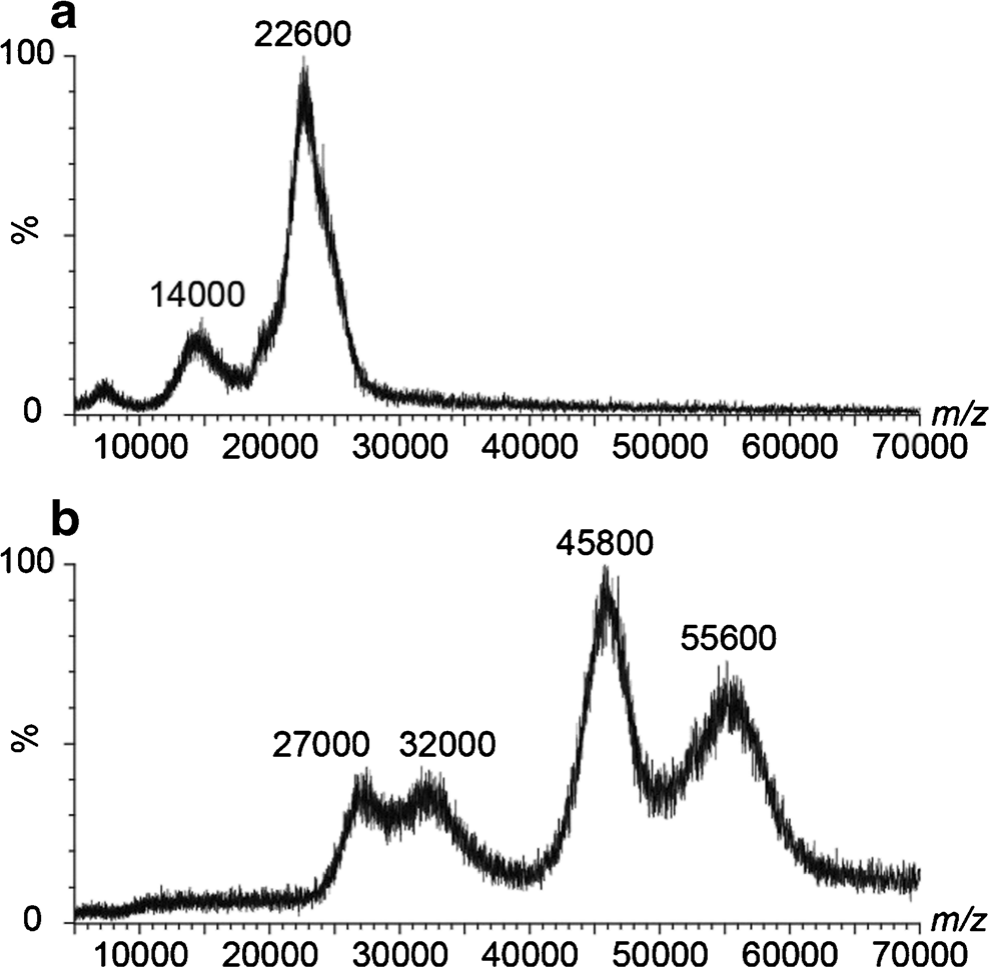
QTOF native ESI MS data for VLPs: CPMV at 50 V collision energy (**a**) and bacteriophage P22 at 100 V collision energy (**b**) are shown. Although in both cases signals are detected, analyte heterogeneity precluded charge state resolution. *m/z* values at peak apices are given. VLPs were exchanged to 40 mM NH_4_OAc, pH 7.0, using 10 kDa M_W_ cutoff filters. Peaks at 14,000, 27,000, and 32,000 *m/z* may represent metastable ions

Besides information on the surface-dry VLP size and an approximation on the bionanoparticle number concentration, two other pieces of information could be gathered from native nES GEMMA spectra. (i) Especially for norovirus West Chester VLPs (Fig. 1b), detection of material below 10 nm EMD hinted the presence of free proteins. Pogan et al. [13] showed that these particles already disassemble at neutral pH and low ionic strengths, which is in line with our results. Moreover, general particle rupture in the nES process is unlikely, as such peaks were only recorded for norovirus West Chester VLPs. (ii) Especially for bacteriophage P22-based VLPs (Fig. 1c) several additional species with significantly lower abundance than the main VLP peak were detected (e.g., at 34 or 45 nm EMD). If these peaks correspond to species simply carrying a higher number of charges from insufficient charge equilibration in the bipolar atmosphere of the nES unit or are analytes of biological relevance (e.g., capsids losing subunits) cannot be determined based on obtained native nES GEMMA results alone. However, ESI ToF MS also showed at least one additional species, which indicates that the observed peaks correspond to different assemblies present in solution (Fig. 2).

### Native ESI MS analysis of VLPs

Next to targeting these VLP analytes via native nES GEMMA, we also applied native ESI MS in VLP characterization. Employing Q-ToF instruments, challenges of VLP M_W_ determination in native ESI MS become obvious. For the same samples as with native nES GEMMA, clear peaks were obtained via native ESI ToF MS (Fig. 2). However, the lack of charge state separation hampered exact mass determination. Common sources for lack of resolution are intrinsic VLP heterogeneity mostly on protein level, e.g., the presence of truncated protein or sequence variants, or simply size resulting in overlapping charge states. As was shown in a previous study, the problems in charge state resolution can be instrument, but as well analyte derived [3]. Furthermore, incomplete desolvation can additionally cause peak broadening, which influences native ESI ToF MS to a higher extent than gas-phase electrophoresis. Nevertheless, using an experimentally derived equation [57], also in such cases, the M_W_ can be estimated from the obtained *m/z* values*.* Taking for instance *m/z* of 22,600 for CPMV into consideration, a M_W_ of 3107 kDa is obtained, for bacteriophage P22, an *m/z* of 55,600 yields a M_W_ of 18,807 kDa. Both M_W_ values are in the same range as data found in literature (see Table 1). In general, the spectra show that the number of different observed sizes is in line with the nES GEMMA results. Moreover, the norovirus VLPs were also analyzed on an ESI CDMS instrument to provide M_W_ values without the need for charge state resolution and obtain more values for the correlation (Electronic Supplementary Material (ESM) Fig. S1). Additionally, application of an Orbitrap instrument with higher resolution might help to resolve charge states in a future study.

### Combining native nES GEMMA and native ESI MS data yields an EMD/M_W_ correlation for VLPs

**Fig. 3:**
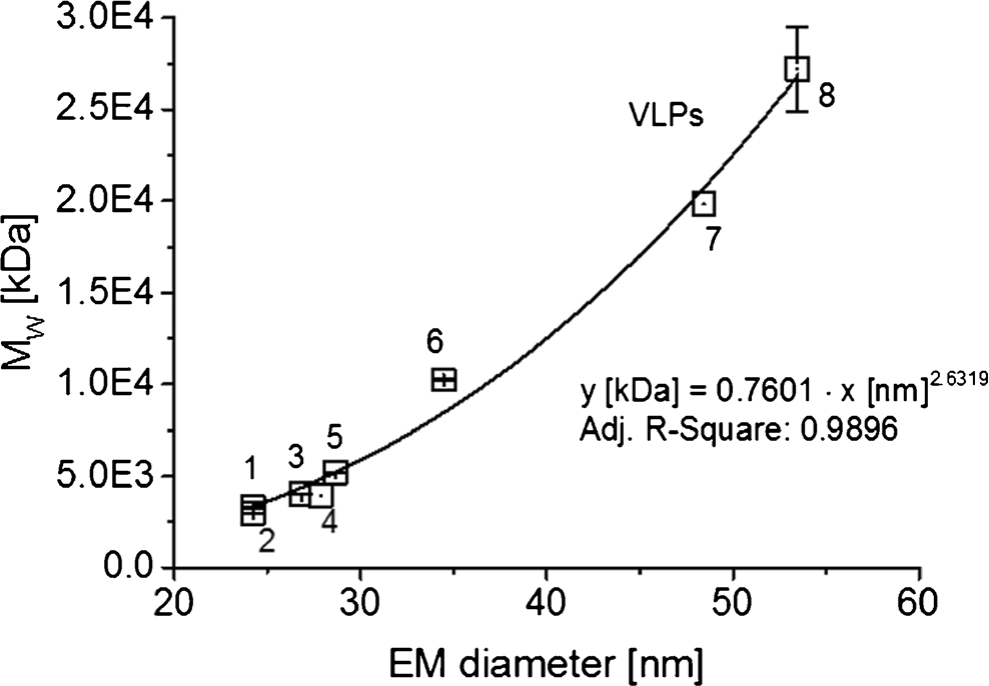
nES GEMMA and MS data can be related to yield an EMD/M_W_ correlation valid for VLPs. The numbering of data points correlates to Table 1

Based on our analyses of VLP material via native nES GEMMA and accurate mass values from native ESI MS including literature values (given in Table 1), we set up a corresponding EMD/M_W_ correlation: y [M_W_ in kDa] = 0.7601 [EMD in nm]^2.6319^ (Fig. 3). Notably, this deviates from a correlation for proteins as was the case for filled virions [32]. Hence, a basic knowledge concerning the analyte class prior EMD-based M_W_ calculation (but not for gas-phase electrophoresis itself) is necessary.

A collapse of VLP particles during gas-phase electrophoresis upon stripping of solvent molecules from their interior seems highly unlikely as AFM and dot-blot analyses of a vaccine VLP demonstrated particle integrity after gas-phase electrophoresis [44]. Instead, due to protein analyte inherent M_W_ limitations, especially a direct comparison of a protein correlation with an EMD/M_W_ correlation for VLPs is to date not feasible. Simply because VLPs are analyzed in an EMD/M_W_ range, in which pure protein complexes (in a non-aggregated or structured form) rarely exist, the extrapolation of the protein EMD/M_W_ correlation to larger EMD and M_W_ values has to be taken with extreme caution. It is of note that the largest protein analyzed in [32] was the octamer of β-galactosidase with an EMD of 16.83 nm and a M_W_ of 931.28 kDa.

In contrast, the VLP-based EMD/M_W_ correlation is based on data points for larger EMD/M_W_ values. VLPs with low EMD/M_W_ value are not reasonable as the proteinaceous sphere has to be of a certain lower size limit (around 20 nm) in order to allow for genome encapsulation within the capsid. Therefore, there is poor overlap between pure protein complex and VLP curves. Extrapolation of either the protein correlation to large or the VLP correlation to lower EMD/M_W_ values is problematic. Hence, we advise against taking one single EMD/M_W_ correlation for all investigated analyte classes in order to calculate M_W_ values based on a particle EMD.

### EMD/M_W_ correlations on different native nES GEMMA instruments

As it was our intention to setup an EMD/M_W_ correlation for VLPs applicable to as many as possible corresponding native nES GEMMA instrumentations, we asked ourselves, if obtained results can be ported between setups. Laschober and colleagues already reported in 2007 differences of up to 15% in obtained EMD values for globular proteins up to 660 kDa [58]. Especially slight variations in nDMA geometries, length values of connecting tubes between instrument parts or differences in sheath flow values may lead to deviations observed between instruments. Therefore, we analyzed a set of analytes on another gas-phase electrophoretic setup besides our standard native nES GEMMA (instrument A). This instrument corresponded to a next-generation setup with differences in the geometry of the nES source and a soft X-ray source for charge equilibration (instrument B). Detection on the latter instrumentation was carried out on a water-based CPC. We opted for immunoglobulin G (IgG), β-galactosidase (β-Gal), several polysaccharides (dextrans and oat β glucans), CPMV VLP, and bacteriophage P22 VLP as analytes.

**Fig. 4:**
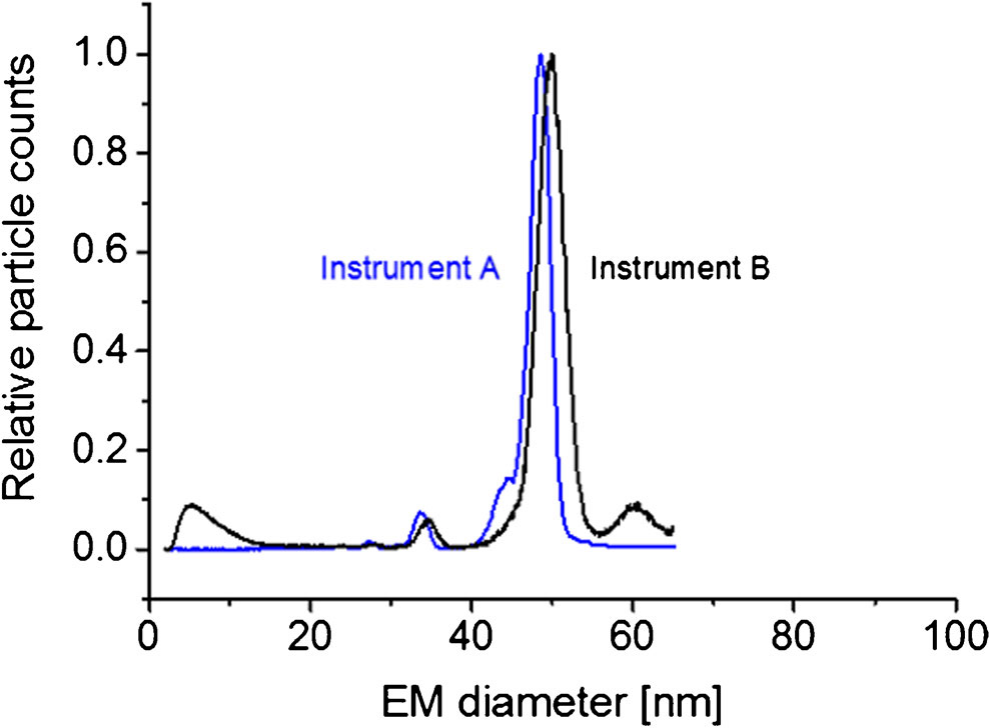
Comparing gas-phase electrophoresis data obtained on two instrument generations. As shown for bacteriophage P22, a significant shift in obtained EMD values on both instruments is found. Corresponding EMD data is found in Table 2

Resulting spectra from gas-phase electrophoresis carried out on the two instrumentations are depicted in Fig. 4 as exemplified by P22 VLPs. Corresponding data for all analytes is given in Table 2. As can be seen, indeed slight differences between instrumentations were detected. For instance, the EMD of investigated proteins deviated on average by 4.5% at the peak apex between our standard instrumentation (instrument A) and the next-generation setup (instrument B). Less variation was found for VLPs, more for polysaccharides. Based on this data, we strongly suggest calibration of each instrument for corresponding EMD-based M_W_ calculation: Instrument specific parameters have to be regarded in order to obtain reliable EMD-based M_W_ values via gas-phase electrophoresis. A simple porting of EMD/M_W_ correlations between instrumentations without considering a corresponding deviation would lead to significant systematic errors in EMD-based particle M_W_ calculation.

**Table 2:** Comparison of averaged EMD values obtained on several gas-phase electrophoretic instrumentations

Analyte		MW (kDa)	Instrument A			Instrument B	
			EMD (nm)	STDEV	(%) value instrument B	EMD (nm)	STDEV
IgG	M	147.27	9.03	0.10	95.19	9.49	0.04
IgG	D	294.54	11.26	0.10	95.58	11.78	0.04
β-Gal	M	116.41	8.33	0.11	95.76	8.70	0.03
β-Gal	D	232.82	10.57	0.11	95.52	11.07	0.02
Dextran 150	M	147.6	8.17	0.15	98.67	8.28	0.01
Dextran 670	M	667.8	10.05	0.41	92.88	10.82	0.12
Oat β glucan 80	M	81	7.12	0.04	93.81	7.59	0.03
Oat β glucan 1500	M	1508	7.71	0.15	94.83	8.13	0.04
CPMV VLP	M	3940	27.88	0.04	99.32	28.07	0.07
P22 VLP	M	19,840	48.44	0.12	97.21	49.83	0.07

At least *N* = 3 measurements were considered per EMD value. M_W_ values and data for instrument A either taken from [32] or Table 1. *M* monomer, *D* dimer; errors provided are standard deviations

### Application of the developed EMD/M_W_ correlation in VLP research, an example

**Fig. 5:**
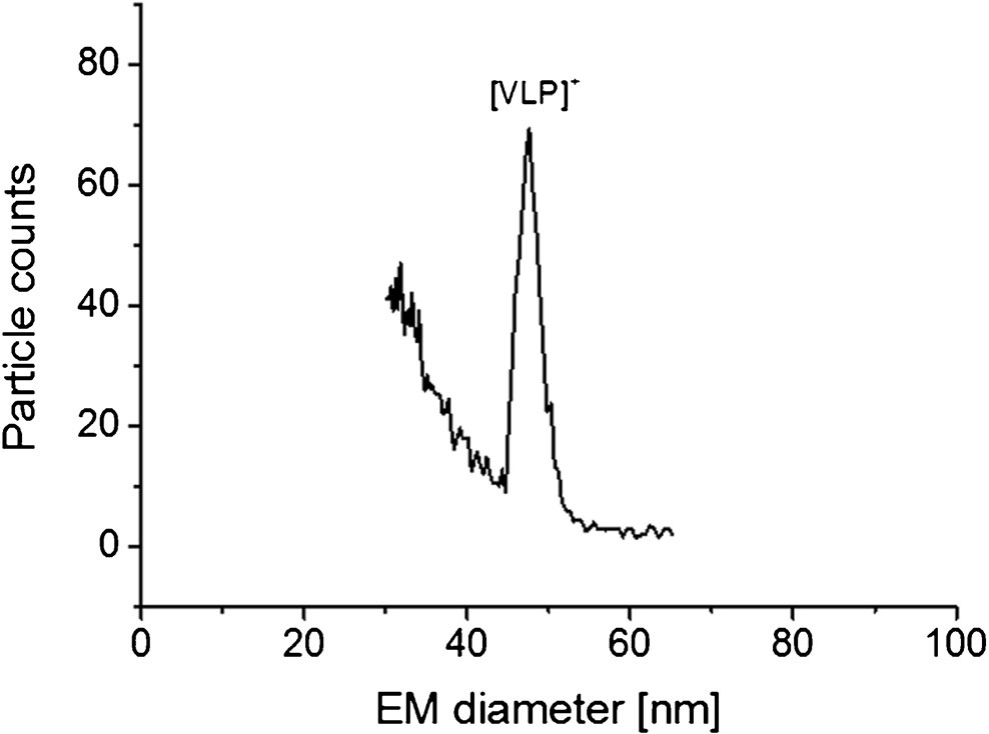
nES GEMMA yields a peak for HPV16-based VLPs allowing its subsequent M_W_ determination based on the correlation presented in Fig. 3. The calculated M_W_ (19,975 kDa) is in good accordance with the theoretically expected M_W_ value (20,260 kDa, based on VLP stoichiometry and database M_W_ values for individual viral proteins)

Following the setup of our EMD/M_W_ correlation for VLPs, we turned to another VLP based on HPV16, for which no native MS data was obtained so far. We carried out our analyses on our standard instrumentation (instrument A). Employing native nES GEMMA, we could obtain a peak at 47.78 ± 0.29 nm EM diameter (*N* = 4 measurements, Fig. 5). Subsequently, we employed our EMD/M_W_ correlation for the calculation of the VLP M_W_. A result of 19,975 kDa is in good accordance with the expected value of 20,260 kDa (based on 72 pentamers of coat protein L1, Uniprot data, P03101, retrieved on January 17^th^, 2019); the deviation is in fact below 1.5%. Reasons for this deviation could be inter alia (i) a still relatively low number of data points available for the VLP EMD/M_W_ correlation, (ii) the shape, surface texture, or tightness of the proteinaceous shell itself, (iii) additional material encapsulated within VLPs, or (iv) differences between VLP material measured via native nES GEMMA and material described in the database. Nevertheless, employing HPV16 as an exemplary VLP bionanoparticle, we were able to demonstrate the applicability of native nES GEMMA-based M_W_ determination of VLP analytes.

## Concluding remarks

For pharmaceutical applications as pointed out above, the thorough characterization of VLPs in terms of particle size, M_W_, sample, and analyte heterogeneity and particle number-based concentration is a necessary prerequisite. In general, it can be seen that ESI MS, whether from ToF or CDMS instruments, is in good agreement with nES GEMMA in terms of species detected. Hence, ESI MS results can be used to interpret nES GEMMA data of unknown samples. However, although native ESI ToF MS is unrivaled in VLP M_W_ determination, it often experiences problems due to sample specific problems, like heterogeneity or low particle numbers. Even though ESI CDMS is not suffering from sample heterogeneity, it is slow, requiring several hours per mass spectrum, and the home-built instrumentation is not widely accessible. nES GEMMA on the other hand is less prone to the mentioned sample inherent characteristics and is relatively cheap facilitating wide application. Analytes are separated according to electrophoretic principles in the gas-phase at ambient pressure based on their size yielding particle number-based concentrations. As has already been shown for other analyte classes, a subsequent correlation between the nES GEMMA-derived EMD and the particle M_W_ allows (bio-)nanoparticle M_W_ calculation in good approximation. We now focused on spherical VLPs and analyzed a variety of these bionanoparticles to setup an EMD/M_W_ correlation, which we found significantly different from correlations known for, e.g., proteins or intact virus particles. As such, it is crucial to know the nature of samples prior to M_W_ determination. As demonstrated, application of this correlation allowed us to calculate the M_W_ of a VLP, for which native ESI MS data is not available to date. Especially through the combination of both methods, nES GEMMA and native ESI MS, as exemplified here, a thorough VLP characterization will be feasible in the future.

## Electronic supplementary material

ESM 1 (PDF 197 kb)
