# Supplementary material for: Virus-like particle size and molecular weight/mass determination applying gas-phase electrophoresis (native nES GEMMA)
Source: Anal Bioanal Chem. 2019 Jul 6;411(23):5951–62. doi: 10.1007/s00216-019-01998-6 (PMC6706367; doi:10.1007/s00216-019-01998-6)
Supplement: Supplementary file 1 — (PDF 197 kb) [file 216_2019_1998_MOESM1_ESM.pdf]

Analytical and Bioanalytical Chemistry

Electronic Supplement Material

Virus-like particle size and molecular weight/mass determination applying gas-phase electrophoresis (native nES GEMMA)

Victor U. Weiss, Ronja Pogan, Samuele Zoratto, Kevin Bond, Pascale Boulanger, Martin F. Jarrold, Nicholas Lyktey, Dominik Pahl, Nicole Puffler, Mario Schelhaas, Ekaterina Selivanovitch, Charlotte Uetrecht, Günter Allmaier

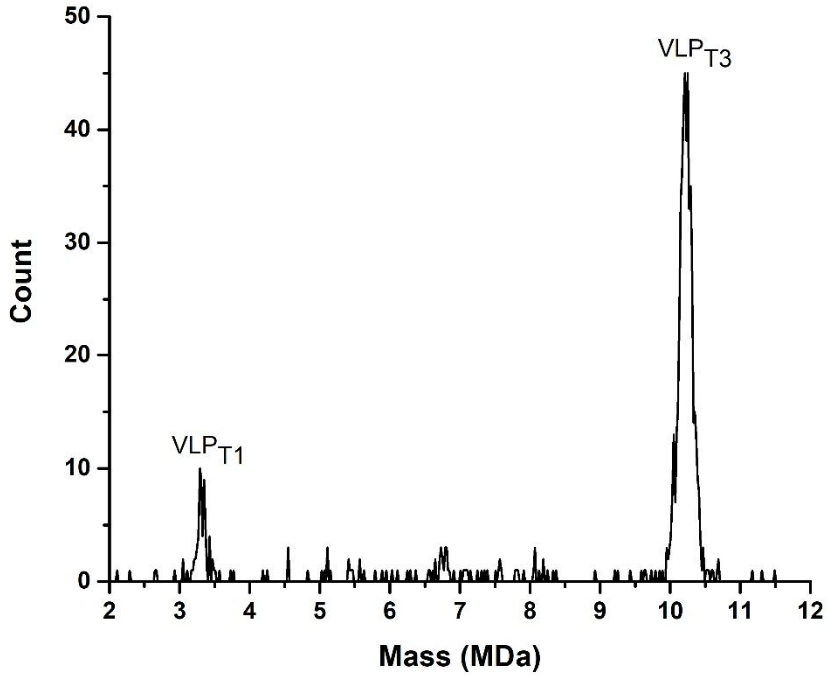

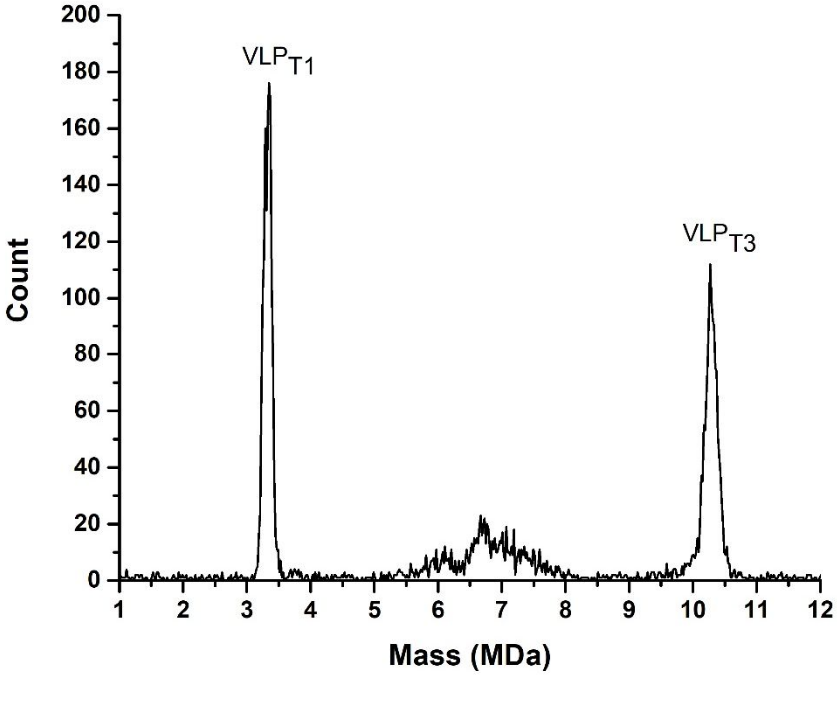

Fig. S1 CDMS spectrum of norovirus West Chester VLPs measured in 50 mM ammonium acetate, pH 6 (top) and 250 mM ammonium acetate, pH 6 (bottom) with peak fitting. 20 kDa bin size were used. Note the overall lower count in the top spectrum indicative of suboptimal spray, which could cause the altered ratios of T1 to T3. Moreover, it is well known that norovirus assembly is sensitive to ionic strength [1, 2]

References

1. Pogan R, Schneider C, Reimer R, Hansman G, Uetrecht C. Norovirus-like VP1 particles exhibit isolate dependent stability profiles. J Phys Condens Matter. 2018;30(6):064006.

2. Shoemaker GK, van Duijn E, Crawford SE, Uetrecht C, Baclayon M, Roos WH, Wuite GJ, Estes MK, Prasad BV, Heck AJ. Norwalk virus assembly and stability monitored by mass spectrometry. Mol Cell Proteomics. 2010;9(8):1742-51.
